# Does Eco-Compensation Alleviate Rural Poverty? New Evidence from National Key Ecological Function Areas in China

**DOI:** 10.3390/ijerph191710899

**Published:** 2022-09-01

**Authors:** Bingtao Qin, Yongwei Yu, Liming Ge, Le Yang, Yuanguo Guo

**Affiliations:** 1 Business School, University of Shanghai for Science and Technology, Shanghai 200093, China; 2 School of Urban and Regional Sciences, Shanghai University of Finance and Economics, Shanghai 200433, China

**Keywords:** poverty alleviation, ecological compensation, synthetic control method, spatial spillovers, Transfer Payment Policy of National Key Ecological Functional Areas

## Abstract

The Transfer Payment Policy of National Key Ecological Functional Areas (TPEFAP), a well-known ecological compensation (eco-compensation) scheme in China, has been proposed by the government to alleviate ecological poverty and protect the environment. In literature, the effectiveness of the TPEFAP on environmental conservation has been widely examined, while few pay attention to the effect of the TPEFAP on poverty alleviation, especially with the consideration of its spatial spillovers as well. In this paper, we utilize panel data covering the key ecological functional areas of China during the period 2011–2018 to evaluate the impact of the TPEFAP on poverty alleviation and also its spatial spillovers by employing the synthetic control method (SCM) and the dynamic spatial Durbin model, respectively. Specifically, we apply the entropy weight method (EWM) to calculate the multidimensional poverty index (MPI) and measure pro-poor effect in terms of MPI change. The results show that: (1) TPEFAP has stable positive effects on MPI in Hubei, Yunnan, Jilin, Gansu, and Ningxia, while the impact on Qinghai fluctuates. (2) MPI presents a significant spatial correlation. Furthermore, both the direct and indirect effects of TPEFAP on MPI are significant and stable positive, for both short- or long-term. (3) For potential channels, rural non-farm employment, rural labor mobility, and agricultural productivity are the key pathways through which the TPEFAP can alleviate poverty both in local and adjacent provinces. However, it is difficult to find significant positive spatial spillovers for the TPEFAP if only the natural resources scale is considered. This study indicates that the government should pay attention to the policy expectations of ecological poverty alleviation and, in future eco-compensation, must further increase the coverage of subsidies and diversify the forms of subsidies.

## 1. Introduction

In 2015, the United Nations identified green development and poverty reduction as the key objectives of “the 2030 Agenda for Sustainable Development” [1], in order to refocus countries’ attentions on ecological restoration and poverty alleviation for global development [2,3]. As the world’s largest developing country, China has made a great economic progress in economy owing to reform and opening up. However, the distribution of reform dividends to all regions is difficult owing to the need to cover enormous territory, making poverty in rural China a major source of concern for the Chinese government. Hence, China’s rapid economic growth resulted in mounting environmental costs and degrading the rural ecosystem [4]. Consequently, rural China simultaneously faces two major issues: environmental destruction and regional poverty [5,6]. Based on the foregoing, China’s State Council issued “the National Plan for Developing Functional Areas” [7] in 2010, which assigns different national land spaces with functions of priority, key, restricted, and prohibited development, and a system of ecological compensation (Eco-compensation) transfer payments for the distribution of restricted and prohibited development areas. Since 2016, the Chinese State Council proposed strengthening ecological compensation and innovating ecological poverty alleviation methods, for coordinating sustainable utilization of the ecosystem and regional development [8]. Therefore, the Chinese government gives ecological compensation policies a new mission, and in 2018, the National Development and Reform Commission and other departments jointly released “the Work Plan of Eco-poverty Alleviation” [9], which clearly states that the role of ecological compensation in poverty alleviation should be improved to achieve a win–win situation for both poverty alleviation and environmental management.

The Chinese government claimed to achieve the goals of poverty alleviation in 2020, which is 10 years ahead of schedule outlined in “the 2030 Agenda for Sustainable Development”. However, only absolute poverty can be addressed in that time. Multidimensional and relative poverty still exist and will become greater problems. Furthermore, despite the alleviation of poverty, severe poverty regions face a high risk of reverting to poverty. This is why the Chinese government is currently working on how to consolidate an early-stage poverty eradication effort and prevent relapse into poverty, and address multidimensional and relative poverty in the future. Therefore, the following questions arise: Can ecological compensation, as a national policy combining poverty alleviation and environmental development in China, solve poverty in rural areas? Simultaneously, owing to disparities in regional factor endowments and actual consequences of policy execution, does ecological compensation have a general poverty alleviation effect? Are there differences in poverty reduction effects between local and neighboring areas? What are the channels through which the policy works towards poverty alleviation goals? And so forth.

**Figure 1:**
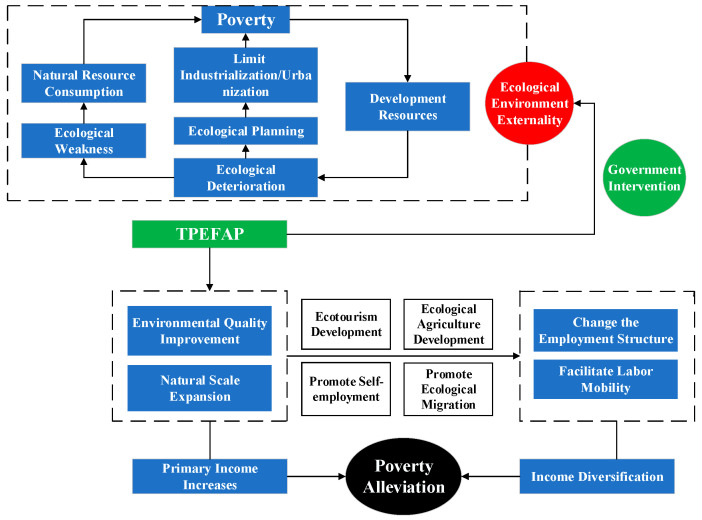
The expected synergism between ecological protection and poverty alleviation under the TPEFAP.

To address these issues, we employ China’s Transfer Payment Policy of National Key Ecological Functional Areas (TPEFAP) as a quasi-natural experiment to investigate the effects of eco-compensation on poverty alleviation and spillover effects. The TPEFAP is one of the largest ecological compensation schemes in China in term of payment, scale, and duration. Initiated in 2008, the TPEFAP originally aimed to alleviate environmental pressure and enhance local ecological quality by relocating local residents and reforesting. However, TPEFAP was opposed by local farmers who face poverty and difficulties in finding alternative livelihoods during implementation. Policy makers began to realize the importance of farmer livelihood issues for the TPEFAP and opened a new round of compensation programs in 2011 (See Table A1). The government assumed that through developing the ecological economy, improving the ecological environment, and providing employment for residents, farmers’ households would be pushed to transform their livelihoods, change traditional production modes, and the externality problem would be internalized (See Figure 1). TPEFAP has the dual goal of protecting ecology and improving livelihoods. Unlike the compensation methods of returning farmland to forest and grass, where subsidies are paid directly to individuals, TPEFAP allocates the subsidies to local governments, which then carry out unified planning and management of the development and construction of key ecological function areas, and this innovating model will maximize the effect of localization.

The TPEFAP has now been implemented for fourteen years and has made significant achievements towards its ecological conservation goal [10]. However, whether the ecological compensation scheme can achieve poverty alleviation has always been discussed by scholars. On the one hand, some scholars argue that ecological compensation was originally intended to improve the environment and, hence, should not be used to reduce poverty [11,12,13]. Research from Mexico and Malawi implies that environmental compensation is far more effective in increasing plant cover than alleviating poverty [14,15]. Wu and Jin [16] point out that under the current compensation standard, the Chinese eco-compensation scheme not only does not improve farmers’ income, but also has a negative long-term impact. On the other hand, most of the rest of scholars believe that the eco-compensation scheme can contribute to poverty reduction [17,18]. Moreover, many scholars empirically show that ecological compensation schemes are feasible for poverty alleviation. Grieg-Gran et al. [19] study the ecological compensation system for forest environmental services in Latin America and find that it substantially raises local farmers’ income levels. Some scholars also claim that ecological compensation reduces poverty by improving socio-economic inequality based on economic models [20]. Several scholars support their view, such as Alix et al. [21], who uses an experimental design to determine the significant effects of PES on social capital in Mexico. Furthermore, a large body and still evolving literature show that, in developing countries, subsidies to farmers can effectively alleviate capital constraints and bring positive welfare effects [22,23]. Subsequent studies consistently confirm these findings, through a serious of field designs from Kenya, Afghanistan, Burkina Faso, and China, which find that the direct or indirect subsidies to farmers can have an improving effect on their consumption levels, liquidity constraints, and household livelihood capital [24,25,26,27]. In addition to focusing on the impact of policy on local poverty, the spillover effects of the policy to reduce poverty have received attention from many scholars [28,29]. Research from United States indicates that policies such as improving employment prospects and investing in social capital can improve spillover effects on poverty alleviation [30]. Palmer-Jones et al. [31] and Wang [32] point out that the spillover effects of poverty reduction policies are more pronounced in developing countries. Further research adopting inter-provincial panel data for 30 Chinese provinces from 1999–2014 reveals that China’s rural financial inclusion policy can affect poverty reduction owing to its spatial spillovers [33]. However, the existing studies do not comprehensively explore the impact of ecological compensation schemes on poverty in neighboring areas and are insufficient to demonstrate the effects of the TPEFAP on the surrounding areas’ poverty. Based on the above studies, the impact of the TPEFAP on rural poverty is still unclear. Whether it contributes to surrounding areas’ poverty reduction requires deeper investigation.

The closest study to us is by Wu et al. [34] who employ a difference-to-difference (DID) model to evaluate the poverty alleviation impacts of an eco-compensation scheme in China. They conclude that the scheme affects significant livelihood improvement among local farmers. However, one difference from our case, in addition to the empirical model, is that their research ignores the scheme’s spatial spillovers. Another related study by Pan and Tang [10] finds significant effects of TPEFAP on water pollution in China.

We try to contribute to existing research in the following ways:

(1) Most studies consider only the spillover effects of other poverty alleviation policies, and few pay attention to the spatial spillovers of eco-compensation.
(2) Most of the existing literature focuses on the environmental improvement effects of ecological compensation and neglects their relationship with poverty, not to mention that there would be studies evaluating the impact of ecological compensation on poverty from different dimensions.
(3) Ecological poverty alleviation assessments in the study areas are mainly confined to valuation in a specific area or individual area, with a lack of studies for a continuous area.
(4) The DID model is always used by studies to assess the relationship between eco-compensation and rural poverty. However, these studies do not consider the potential endogeneity of the DID, conversely, the SCM not only overcomes the endogenous problem, but also visualizes the net effects of policy implementation.

The structure of this paper is as follows: Section 2 presents the variables measurement and research methods; Section 3 presents and discusses the empirical results; Section 4 concludes the study; and Section 5 presents corresponding policy implications.

## 2. Methodology

### 2.1. Measuring Method for MPI of the TPEFAP

**Figure 2:**
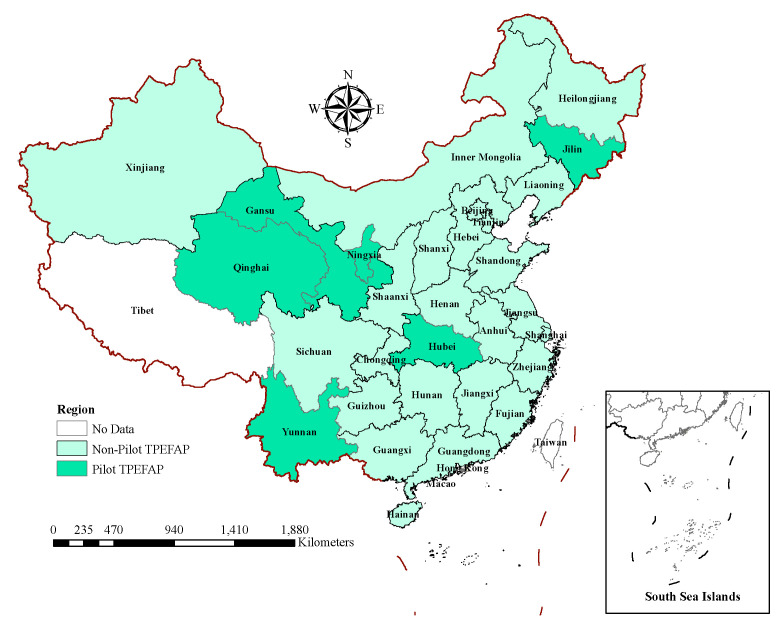
Study area.

The multidimensional poverty serves as the explanatory variable in this paper, as well as the preliminary basis for accurately assessing the poverty reduction effect of TPEFAP based on its reasonable construction. Considering the specific situation of the areas receiving TPEFAP subsidies, therefore the reasonable construction of MPI is closely related to the selection of study area. In this paper, we take the number of counties receiving TPEFAP compensation in a province as a benchmark, and if it is greater than 1/3, then it becomes the target province of this study, and if not, then it is excluded. (As a benchmark, 1/3 is justified for following reasons: (1) TPEFAP implementation is based on the smallest unit of counties, and our study starts at the inter-provincial level. Therefore the number of subsidized counties in the target provinces must be of a certain size, and rural areas are concentrated in these counties, and 1/3 is precisely in line with the actual ratio of one poor county for every three counties in China’s underdeveloped provinces; (2) from the data we obtained on TPEFAP subsidized counties, we estimate that there are over 1/3 subsidized counties in the six provinces we chose, while the other subsidized counties are spread out throughout the provinces, so 1/3 allows for a more realistic cut-off point that excludes more provinces). After careful deliberation, we chose six provinces with the longest implementation period and the largest coverage, namely Jilin, Hubei, Yunnan, Ningxia, Qinghai, and Gansu (See Figure 2), which are mainly concentrated in northwestern and southwest China, and thus also fit the characteristics of key ecological function areas with abundant natural resources but low environmental carrying capacity and backward economic development.

Finally, in light of “*the Outline for Development-Oriented Poverty Alleviation for China’s Rural Areas (2011–2020)*” [35], and considering the actual development goals of above six provinces, this paper attempts to build an MPI to measure poverty levels in four dimensions: education, medical, economy, and ecology. The MPI is indicated in Table 1.

Based on Table 1, we adopt the entropy weight method (EWM) to calculate the above index weights, and the specific calculation process was described by Li et al. [36]. As an objective method, the EWM determines the weight size based on the index’s inherent properties and degree of variation, which can reduce the influence of subjectivity. Meanwhile, the polyhedron method, polygon method, vector sum method, and weight sum method are all equivalent to measure multidimensional poverty [37,38], so we chose the most widely used weight sum method to calculate MPI [39,40,41], calculated as follows:

(1) $$MPI=\omega_{1}\times M_{1}+\omega_{2}\times M_{2}+\omega_{3}\times M_{3}+\omega_{4}\times M_{4}$$

In Equation (1), MPI represents the multidimensional poverty level of ecological function areas; $M_{1},M_{2},M_{3},M_{4}$ represent education, medical, economy, and ecology, respectively; and $\omega_{1},\omega_{2},\omega_{3},\omega_{4}$ represent the weights of the corresponding dimensions, respectively. (It is important to note that the EMW should determine the positive and negative direction of each indicator, and in this paper, we used the Engel coefficient of rural residents as a negative indicator, which means that the higher the MPI is, the richer it is, and the lower it is, the poorer it is).

### 2.2. Synthetic Control Method

The SCM proposed and refined by Abadie and Gardeazabal [42] is suitable for policy evaluation type studies. Additionally, compared to traditional policy evaluation methods such as DID, SCM can be effective in overcoming endogenous problems which may be caused by factors such as selection bias in the selection of control groups, and can isolate the effects of TPEFAP that reduce poverty more objectively and accurately. Therefore, we attempt to employ the SCM to estimate the 2006–2018 panel data of Yunnan, Gansu, Ningxia, Hubei, Jilin, and Qinghai.

It is supposed that there are provinces i=1,⋯,N in $t=1,\cdots T_{0},\cdots T$ period, and $T_{0}$ is the opening time of TPEFAP, namely 2011. Consider $Y_{0it}$ as the MPI of key ecological function areas which are not affected by TPEFAP. Consider $Y_{1it}$ as the MPI of i province at t period which is affected by TPEFAP. Therefore, $t=1,\cdots ,T_{0}$ province’s MPI is not affected by TPEFAP, and $Y_{0it}=Y_{1it}$. After the implementation of TPEFAP ($t>T_{0}$), $\beta_{it}=Y_{1it}-Y_{0it}$ represents the poverty-reduction effect of TPEFAP. Additionally, if the province without TPEFAP, $Y_{0it}$ is a known parameter, $Y_{1it}$ is unknown, and synthesizing the ‘counterfactual’ $Y_{1it}$ is necessary. For TPEFAP provinces, $Y_{0it}$ is an unknown parameter, and $Y_{1it}$ is known. The Equation (2) can be used to estimate this counterfactual result [43]:

(2) $$Y_{0it}=\delta_{t}+\theta_{t}Z_{i}+\lambda_{t}\mu_{i}+\varepsilon_{it}$$

In Equation (2), $\delta_{t}$ represents the time fixed effects, $Z_{i}$ is a vector of control variables unaffected by TPEFAP, namely Labor,Urban,ATFP,Natural,Gov,Pgdp and Invest, $\lambda_{t}$ is a (1×F) vector of unobservable common factor, $\mu_{i}$ is a 1×F vector of unobservable regional fixed effects, and $\varepsilon_{it}$ is the error term.

It is assumed that i=1 is a TPEFAP province (Yunnan, Gansu, Ningxia, Hubei, Jilin or Qinghai), and the rest of N provinces (24 control group provinces) are unaffected by TPEFAP. Consider a N×1 vector $W=(w_{2}\cdots w_{N})$ where $w_{j}\geq 0,j=2,\cdots ,N+1,\;w_{2}+\cdots +w_{N+1}=1$, and each vector of W represents a virtual synthetic control combination. Weighing the variables for each control group gives us Equation (3):

(3) $$\sum_{j=2}^{N+1}w_{j}Y_{jt}=\delta_{t}+\theta_{t}\sum_{j=2}^{N+1}w_{j}Z_{j}+\lambda_{t}\sum_{j=2}^{N+1}w_{j\mu_{j}}+\sum_{j=2}^{N+1}w_{j}\varepsilon_{jt}$$

Suppose that there is a $W^{*}=(w_{2}^{*},\cdots ,w_{N+1}^{*})$, when $t\leq T_{0}$, such that

(4) $$\sum_{j=2}^{N+1}w_{j}^{*}Y_{j1}=Y_{11}\sum_{j=2}^{N+1}w_{j}^{*}Y_{j2}=Y_{12},\cdots ,\sum_{j=2}^{N+1}w_{j}^{*}Y_{jT_{0}}=Y_{1T_{0}^{\prime }}\sum_{j=2}^{N+1}w_{j}^{*}Z_{j}=Z_{1}$$

Equation (4) has been proved that when $t>T_{0}$ (Abadie and Gardeazabal, 2003) [42]:

(5) $$Y_{01t}-\sum_{j=2}^{N+1}w_{j}^{*}Y_{kt}=\sum_{j=2}^{N+1}w_{j}^{*}\sum_{s=1}^{T_{0}}\lambda_{t}{\left(\sum_{n=1}^{T_{0}}\lambda_{n}^{\prime }\lambda_{n}\right)}^{-1}\lambda_{s}^{\prime }\left(\varepsilon_{js}-\varepsilon_{1s}\right)-\sum_{j=1}^{N+1}w_{j}^{*}\left(\varepsilon_{jt}-\varepsilon_{1t}\right)=0$$

Therefore, when $T_{0}<t\leq T$, the ‘counterfactual’ control group of Yunnan, Gansu, Ningxia, Hubei, Jilin, and Qinghai will be synthesized by other 24 provinces, such that $\hat{Y_{01t}}=\Sigma_{j=2}^{N+1}w_{j}{}^{*}Y_{jt}$. Based on this, we can estimate the policy effect:

(6) $$\hat{\beta_{1t}}=Y_{1t}-\sum_{j=2}^{N+1}w_{j}^{*}Y_{jt},t\in \left[T_{0}+1,\cdots ,T\right]$$

Equation (6) can be considered as the estimator of $\beta_{1t}$, and that is the poverty-reduction effect of TPEFAP we try to estimate.

### 2.3. Dynamic Spatial Durbin Model

The above studies suggest that ignoring TPEFAP’s policy spillovers may result in biased assessment results; therefore, we utilized the spatial panel Durbin model for empirical analysis. By incorporating the TPEFAP dummy variable (Dvv) into the model, we also introduced the time-lag of MPI into the static spatial Durbin model to establish the Dynamic Spatial Durbin model, which takes into account the possible path-dependence of ecological poverty alleviation in the time dimension, and the possible reverse causality between ecological poverty alleviation and economic growth and other factors leading to endogeneity problems:

(7) $$\begin{array}{l} MPI_{it}=\beta_{0}+\beta_{1}MPI_{it-1}+\rho_{1}\sum_{i=1}^{n}w_{ij}MPI_{it}+\beta_{2}G_{i}\cdot D_{t}\\ +\rho_{2}\sum_{i=1}^{n}w_{ij}G_{i}\cdot D_{t}+\lambda \sum_{i=1}^{n}w_{ij}X_{it}+\delta \sum X_{it}+\varepsilon_{it} \end{array}$$

where $MPI_{it}$ represents multidimensional poverty index of i province at t period, while $MPI_{it-1}$ represents i province at t−1 period, $G_{i}$ represents a region dummy variable, where TPEFAP is implemented, $G_{i}=1$, otherwise $G_{i}=0$, and $D_{i}$ is a time dummy variable, when $t\geq 2011,\;D_{t}=1,$ otherwise $D_{t}=0$. Therefore $Dvv_{i,t}=G_{i}\times D_{t}$; w is spatial weight matrix; β,ρ,λ,δ are parameters. In addition, we consider the numerous factors influencing poverty alleviation, and introduce a series of control variables X, including the rural non-farm employment (*Labor*), rural labor mobility (*Urban*), agricultural productivity (*ATFP*), natural resource scale (*Natural*), rural financial expenditure (*Gov*), economic development (*Pgdp*), and rural investment level (*Invest*). For the setting of the spatial weight matrix, existing studies often adopt the adjacency weight matrix, distance weight matrix, economic weight matrix, etc. to reflect the spatial interaction relationship between regions [44,45]. However, we consider that ecological poverty alleviation is not only spatially linked to neighboring areas through geographical distance, but also interacts with them economically. Thus, we construct an asymmetric nested spatial weight matrix ($W_{1}$) to more rigorously examine the impact of TPEFAP on poverty reduction in the surrounding areas, which integrates the combined effects of geographical distance and economic linkages, calculated as follows:

(8) $$W_{1}=W_{d}\mathrm{diag}\left(\bar{Y_{1}}/\bar{Y},\bar{Y_{2}}/\bar{Y},L,\bar{Y_{n}}/\bar{Y}\right)$$

(9) $$\bar{Y_{i}}=\sum_{t_{0}}^{t_{1}}Y_{it}/\left(t_{1}-t_{0}+1\right)$$

(10) $$\bar{Y}=\sum_{i=1}^{n}\sum_{t_{0}}^{t_{1}}Y_{it}/n\left(t_{1}-t_{0}+1\right)$$

where ${\bar{Y}}_{i}$ is the average value of gross output value of agriculture, forestry, animal husbandry and fishery (our study focuses on rural poverty, so we chose agriculture, forestry, livestock, and fishing as the economic factor in the nested matrix, which is more consistent with the research topic) in i province during the TPEFAP implementation period, while $\bar{Y}$ is the average value of gross output value for agriculture, forestry, animal husbandry, and fishery in all provinces during the same period. $W_{d}$ is the geographic distance weight matrix.

### 2.4. The Impact Mechanism Model of the TPEFAP on MPI

We explore this potential channel by inputting the interaction terms of the policy dummy variable (Dvv) with the rural labor structure (*Labor*), rural labor mobility (*Urban*), agricultural productivity (*ATFP*), and natural resource scale (*Natural*) in turn (see Equation (11)).

(11) $$\begin{array}{l} MPI_{it}=\chi_{0}+\chi_{1}MPI_{it-1}+\kappa_{1}\sum_{i=1}^{n}w_{ij}MPI_{it}+\chi_{2}G_{i}\cdot D_{t}+\kappa_{2}\sum_{i=1}^{n}w_{ij}G_{i}\cdot D_{t}+\\ \chi_{3}G_{i}\cdot D_{t}\times Channel+\kappa_{3}\sum_{i=1}^{n}w_{ij}G_{i}\cdot D_{t}\times Channel+\sum X_{it}+\chi_{4}\sum_{i=1}^{n}w_{ij}X_{it}+\delta_{it} \end{array}$$

where *Channel* represents *Labor*, *Urban*, *ATFP* and *Natural* potential mechanisms, $\chi_{0}$ and $\delta_{it}$ in Equation (11) denote the constant term and error term, respectively.

### 2.5. Variable Selection and Data Source

#### 2.5.1. Variable Selection

Multidimensional poverty index (*MPI*) is selected as the outcome variable to represent the poverty improvement of each province. The dummy variable (Dvv) represents whether TPEFAP is opening in a province or whether TPEFAP is the predictor variable.

TPEFAP’s analytical model of the driving mechanism in multidimensional poverty includes core explanatory variables as well as control variables to alleviate estimation bias. We selected four core explanatory variables from different dimensions to explore the impact of TPEFAP on multidimensional poverty: (1) rural non-farm employment (*Labor*). In one respect, after being affected by TPEFAP, some areas will choose to close mountains for afforestation and return farmland to forests for ecological governance, so that those farmers in ecological function areas will either be out of work or will use subsidies for self-employment activities as the result of a reduction in agricultural land and the lower demand for agricultural labor. In another, the government also provided some eco-care positions (e.g., eco-ranger) to increase employment opportunities for local people. (2) rural labor mobility (*Urban*). The key ecological function areas are generally built in ecologically fragile areas, and local farmers will then move to areas with a good ecological environment as a result of the policy, i.e., ecological migrants (There have some specific cases of ecological migration. For example, the area-free ecological migration zone in Taishun County (see http://www.wenzhou.gov.cn/art/2019/8/19/art_1217829_37128223.html, accessed on 19 August 2019); the ecological migration work for the Sanjiangyuan area (see http://www.gov.cn/jrzg/2011-08/05/content_1920163.htm, accessed on 5 August 2011), etc.). The situation will encourage the urbanization of areas benefiting from the TPEFAP subsidies, and urbanization will lead to better education levels and medical facilities, alleviating medical poverty and education poverty. Furthermore, the agglomeration effect caused by labor mobility will further affect the poverty situation in the surrounding areas. (3) Agricultural total factor productivity (*ATFP*). In the process of changing land from agriculture to other uses, the transformation of agricultural productivity to production service productivity may occur, and may benefit from eco-compensation [46]. Concerning TPEFAP, it changes the use of land in key ecological function areas, increasing the value of land use and alleviating economic poverty. (4) Natural resource scale (*Natural*). The relationship between livelihood systems, ecosystems, human well-being, and ecological services is particularly important in the context of eco-compensation [47,48]. For instance, the improvement of the natural environment due to eco-compensation will contribute to the development of ecotourism, while local farmers can benefit from ecotourism’s cultural services and lower economic poverty, and TPEFAP contributes to the improved water quality it brings which can enhance local aquaculture. Additionally, the economic effects caused by the expansion of local natural resources will be imitated by neighboring regions, which will further form a positive interaction between regions.

In addition to TPEFAP implementation, local economic and social development may also impact poverty, so we included the following variables as controls: economic development (*Pgdp*), government expenditure (*Gov*), and rural investment level (*Invest*). In particular, the last three variables are logarithmic in the empirical research (the variable descriptive statistics is shown in Table A2).

#### 2.5.2. Data Source

The data of counties enjoying TPEFAP subsidies selected from 2006 to 2018 was obtained by our manual application from the provincial and municipal finance departments and government websites in China, including whether a county accepted TPEFAP subsidies in a certain year and whether it was classified as a national key ecological function area. Quantitative data on economy, education, medical, and ecology come from the “China Statistical Yearbook”, “China Health Care Statistical Yearbook”, and “provincial educational statistical yearbooks”. Agricultural variables and control variables data are mainly from the “China Rural Statistical Yearbook”, “China Agricultural Yearbook”, and the wind database. Considering the lack of data for Tibet and the poor availability of data for Hong Kong, Macao, and Taiwan, the study is conducted in 30 provinces, excluding Tibet, Hong Kong, Macao, and Taiwan. Moreover, to eliminate the impacts on changes in general price, the GDP series have been converted to the constant 1978 price.

It is worth mentioning that since the data about TPEFAP in China are not publicly available, we make an on-demand disclosure to the relevant provincial departments and ultimately collect all the data of 30 provinces over several months, which are reliable, authentic, and authoritative.

## 3. Results

### 3.1. Synthetic Control Results

Finally, the MPI data of Yunnan, Gansu, Ningxia, Hubei, Jilin, and Qinghai were synthesized by SCM. Table 2 depicts the weights of each control province in the six synthetic provinces. The weights indicate that the MPI of Yunnan, was best synthesized by a combination of Guangdong, Guizhou, and Xinjiang. Similarly, Gansu is best synthesized by Shanghai, Henan, Hainan, Guizhou, and Xinjiang. The specific weights table of these provinces are displayed in Table 2.

**Figure 3:**
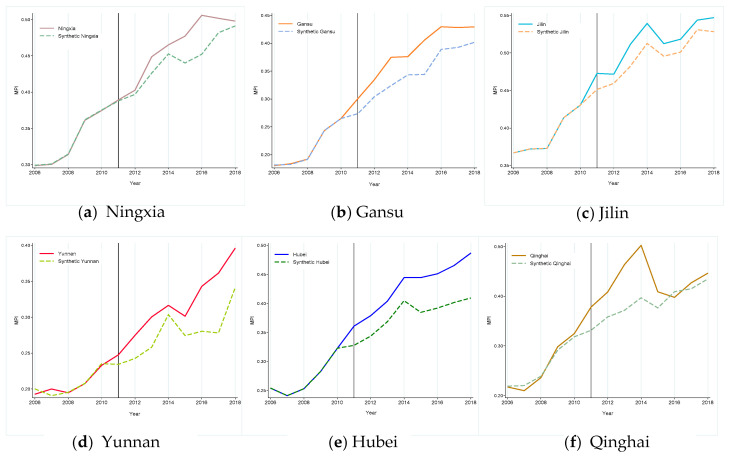
Comparison of MPI in the observed provinces and synthetic provinces from 2006 to 2018. Note: The black vertical solid line represents the opening of TPEFAP, the dashed curve is a synthetic MPI, and the solid curve is the actual MPI, the same below.

Figure 3 plots the synthetic and observed MPI flow for six target provinces from 2006 to 2018. Where the vertical solid line indicates time of full TPEFAP implementation (in 2011), the solid and dashed curve represent the MPI develop track for the observed and synthetic provinces, respectively, and the gap between the two show net policy effect of TPEFAP implementation. As shown in Figure 3, before the opening of TPEFAP (2006–2010), the solid and dashed curve almost overlap, which proves that the SCM can synthesize the track of development for these provinces’ MPI. After the opening of TPEFAP, the synthetic provinces curve is obviously under the observed provinces curve, which means that TPEFAP had a strong positive effect on the poverty alleviation in Yunnan, Gansu, Ningxia, Hubei, Jilin, and Qinghai.

After the vertical solid line, the positive effect of TPEFAP on six provinces’ MPI present upward movement of varying degrees (although the synthetic Qinghai curve pilot intersected with observed Qinghai curve in 2016, the overall trend is upward). Specifically, the poverty alleviation effects are most significant in Hubei, Yunnan, and Gansu, while the policy effects are relatively weak in Qinghai. This may be because. (1) As the only pilot in central China, Hubei province had a broad-based policy approach and was in the early stages of eradicating poverty, thus the TPEFAP effect on Hubei is immediate; (2) Due to its geographical location in the southwest border of China, where poor mountainous areas crisscross all regions, the Chinese government has focused on Yunnan province by giving more subsides to TPEFAP, which makes the MPI of Yunnan kept increasing year by year. (3) similarly, Gansu is located in northwest China, where a scarcity of resources leads to a serious exodus of labor, resulting in deep poverty, but as mentioned in a theoretical framework, the opening of TPEFAP may alleviate this situation by improving the development of environment and related industries, and eventually attracting some of the labor force back. (4) While Qinghai has the largest land area among six provinces (area of Qinghai Province: 722,300 (km^2^), Hubei = 185,900, Jilin = 187,400, Gansu = 453,700, Yunnan = 394,100, Ningxia = 66,400), it is difficult for TPEFAP dividends to truly benefit the whole province. Furthermore, Qinghai has more poverty agglomerations and a slower industrialization process so it is hard to develop rural employment and industries overnight by injecting TPEFAP subsides, which may explain why there is an intersection of two curves in the province after policy intervention.

In the Ningxia synthesis result of Figure 3a, the observed Ningxia curve obviously exceed the synthetic curve after the vertical solid line, but the observed Ningxia curve shows a downward trend in 2017–2018, which may be related to the decrease in policy inclination and slowdown in subsides growth. (In 2015, Ningxia was compensated by TPEFAP with an amount of CNY 333 million, which increased to CNY 1451 million yuan in 2016, showing a funding growth rate of 335%, while there was a significant decreasing trend with only CNY 155,100 and 157,600 million of funding in 2017 and 2018, respectively). Although Jilin also shows a significant poverty alleviation effect, the observed Jilin curve fluctuates to some extent after the vertical solid line; the possible explanation is that Jilin, as one of China’s three northeastern provinces, suffers a lag in economic development after China’s transition from industrialization to information technology. TPEFAP’s implementation may temporarily eased the poverty of local farmers, nevertheless the decay of the local industrial sector and the economic slowdown would partially offset the pull effect of TPEFAP.

### 3.2. Results of Robustness Test

#### 3.2.1. Result of Permutation Test

**Figure 4:**
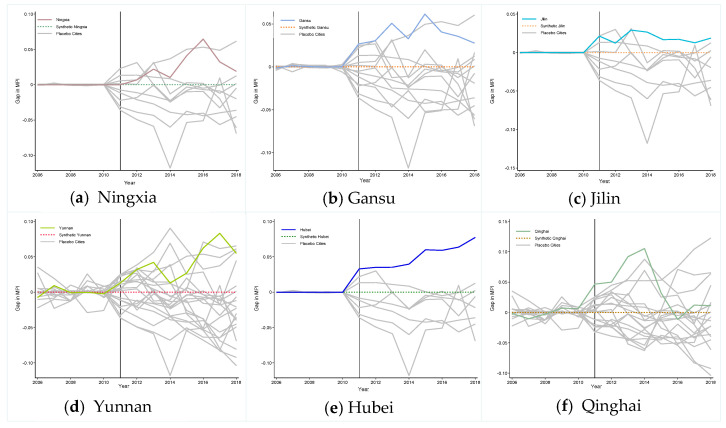
Permutation Test-MPI gaps in six treated provinces and placebo gaps in donor provinces.

As shown in Figure 4, the bolded curve and gray thin curve represents the prediction error in treated provinces and synthetic provinces, respectively. It can clearly be observed these curves are around zero before the vertical solid line, indicating that the synthetic provinces can perfectly match the pre-intervention MPI of treated provinces. After vertical solid line, the gap for the bolded curve is higher than a gray thin curve, indicating that if we randomly select a control unit, there is a small probability that we will obtain a result consistent with the target province. That is to say, it is effective to employ the SCM to estimate the effect of TPEFAP on poverty alleviation in key function areas. It is worth mentioning that Qinghai’s bolded curve begins to decline after 2016, which coincides with the description in Figure 3f, the possible reasons are the large area of Qinghai province, the number of poor areas, etc., as explained above.

#### 3.2.2. Result of Placebo Test

**Figure 5:**
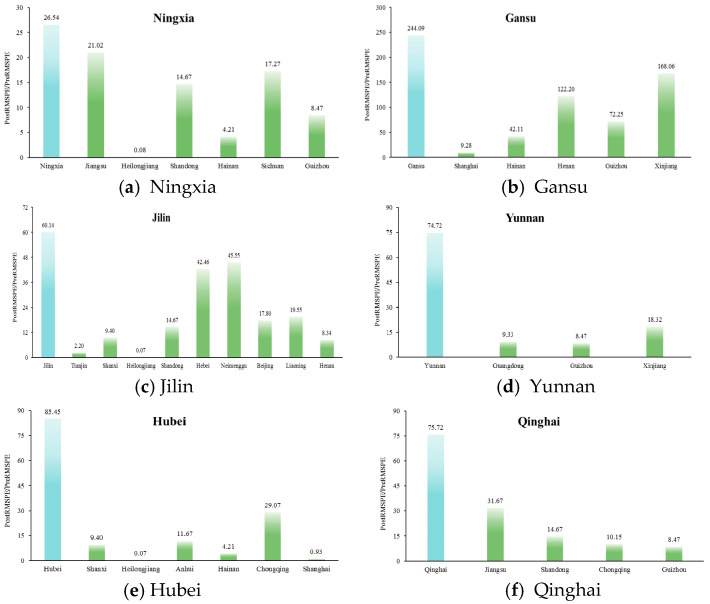
Distributions of the ratio of RMSPE before and after TPEFAP for six provinces.

As shown in Figure 5, RMSPE ratios for the six treatment provinces are 26.54, 244.09, 60.14, 74.42, 85.45, and 75.52 (from a to f) and all higher than the RMSPE ratios of the respective similar control units to varying degrees, which demonstrates the validity of TPEFAP for poverty alleviation.

#### 3.2.3. Result of Iterative Test

**Figure 6:**
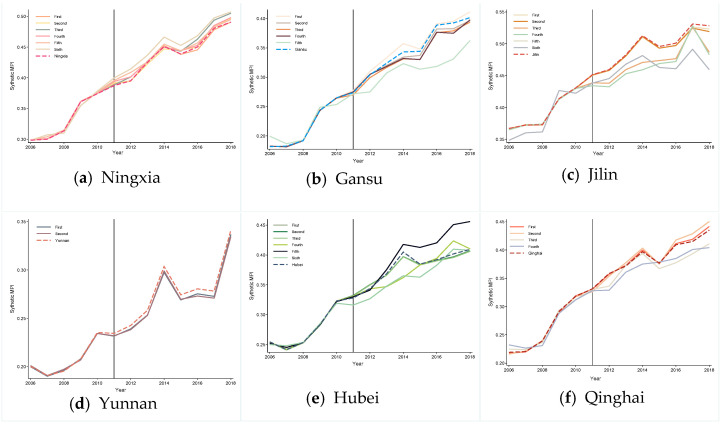
Comparison of MPI development tracks after removing donor groups in successive iterations.

Figure 6 plots after eliminating provinces with non-zero donor pool weights one by one. (We removed provinces with large donor pool weights, i.e., provinces in the control group with high contribution). The iterative synthetic provinces tracks are very close to the treated provinces tracks, and they almost overlap in some intervals, indicating again that the robustness of employing the SCM to assess TPEFAP effects does not vary with donor group.

### 3.3. Spatial Regression Results

#### 3.3.1. Direct Effect Estimation Result 

The result of the Global Moran’s Index in MPI is strongly statistically significant (above 1%) each year, indicating that the application of the spatial econometric model to analyze the data is necessary (see Table A3). Additionally, both the Wald and LR tests are significant at different levels, which means that SDM is the most appropriate choice for our study (see Table A4).

As shown in Table 3, we find the overall regression results of the non-spatial general panel and non-spatial dynamic panel to be unsatisfactory due to ignoring the potential endogeneity problem or spatial correlation problem. Moreover, a comparison of models 4 and 5 validates the path-dependence of ecological poverty reduction in the time dimension, since the coefficient of MPI time lag term (L.MPI) in Model 5 is significantly positive at the 1% level. Model 5 in Table 4 shows that Dvv has a positive consequence on the development of MPI at a 1% significant level, which indicates that implement the TPEFAP is helpful to alleviate poverty in local provinces. Additionally, the coefficient of Dvv from Model 1 to Model 4 all have a positive effect on MPI at various significant level, which corroborates the robustness of TPEFAP to improve multidimensional poverty in Model 5. From the analysis of spatial lag coefficient, the elasticity coefficient of W×Dvv is 0.015 at a 10% significant level, which shows that TPEFAP implementation has a positive spillover effect on MPI in the surrounding provinces. This may be because: (1) in the context of strategic inter-regional competition for poverty reduction in China, TPEFAP’s results in poverty alleviation in local areas can have a demonstration effect on neighboring provinces, facilitating TPEFAP’s improvement of regional poverty between provinces. (2) Local governments were under pressure to reduce poverty after China’s State Council proposed “comprehensive poverty alleviation” in 2012. By improving the local agricultural technology and upgrading the labor force structure, TPEFAP can develop the MPI of neighboring provinces through multiple channels, such as economic cooperation, labor mobility, and technology spillovers. (3) As officials in non-TPEFAP provinces still face the political task of “poverty eradication”, they were forced to focus on ecological function areas due to the task of reducing poverty and assessing performance.

#### 3.3.2. Decomposition Effect Estimation of Short- and Long-Term Results

Table 4 shows that the direct and indirect effects of TPEFAP implementation on poverty alleviation in local and neighboring areas all are significant in the short- and long-term, which may suggest that the above results are robust. (To understand more specifically the regression coefficients of the spatial lag term in the dynamic spatial Durbin model, we decomposed the total effect of Dvv improving MPI into direct and indirect effects according to the partial derivative matrix estimation method in the short- and long-term). In detail, the indirect and direct effect Dvv coefficients in the short-term are 0.02699 and 0.02067, respectively, which also pass the 1% and 10% significance test, respectively, illustrating that there is an immediate impact of TPEFAP implementation on improving MPI, while the policy spillover channel is relatively smooth. Furthermore, in the long-term, the direct, indirect, and total effects of TPEFAP on MPI improvement are significantly enhanced, increasing by 192.5%, 235%, and 216.7%, respectively, indicating that, with the enhancement of subsides and scope and the regulation of compensation regulations, the poverty-alleviation effect of TPEFAP on key ecological function areas continues to strengthen, while the positive spatial spillovers on surrounding areas are gradually evident. In terms of the change in TPEFAP’s indirect and direct effects (being equal in the short term to be significantly greater in the long term), it shows that TPEFAP’s optimization of the labor structure, labor mobility, and agricultural productivity in local areas gradually improve over time, and its focus will shift to neighboring provinces, thus enhancing positive spillovers.

### 3.4. The Potential Mechanisms Test Result

Table 5 illustrates the following results: (1) in terms of Labor, a lower share of non-farm employment locally suppresses MPI, while a lower share of non-farm employment in surrounding provinces significantly alleviates local poverty. That may support above theoretical analysis, i.e., the higher the proportion of primary production, the lower the degree of employment diversification of farm households, and the less favorable to MPI. In addition, the coefficient of Dvv×Labor is negative at the 1% significance level in Model 6 to Model 9, which indicates the optimization of the rural labor structure has contributed to the TPEFAP effect. From the analysis of spatial lag coefficient, the interaction term coefficient of W×Dvv×Labor all are positive at a 1% significant level as well, which means that through Labor, TPEFAP could indeed improve MPI in the surrounding areas.

(2) From the rural labor mobility (*Urban*) channel, the coefficients of *Urban* and W×Dvv×Urban are 0.236 and 0.922, respectively, which all pass 1% significance test. That shows that as a result of TPEFAP implementation, many farming families have relocated to surrounding more developed provinces, accelerating ecological migration. The implementation of TPEFAP has also improved the MPI of the surrounding areas by bringing much productivity and economic development to those areas.

(3) The effects of ATFP on MPI in both local and adjacent areas are significantly positive, which is consistent with the previous analysis that improving agricultural productivity would lead to the efficiency of farmers’ production activities, and technology spillover would benefit farmers in adjacent areas. Nevertheless, the coefficients of Dvv×ATFP are positive but all insignificant in Model 7 to Model 9, which shows that agricultural productivity gains from TPEFAP may be difficult to translate into poverty reduction in the short term. That may be due to local governments preferring direct poverty reduction based on ecological migration over indirect poverty alleviation based on productivity improvement. Meanwhile, agricultural productivity improvement is a long-term endeavor that requires sustained investment, and it is difficult to show immediate feedback effects.

(4) In Model 6 to Model 9, MPI is significantly impacted by the expansion of local natural resources, while the expansion of natural resources in surrounding areas has a negative spatial spillover on MPI locally. The coefficients of Dvv×Natural are all positive at 1% significant level, which demonstrates TPEFAP has effectively improved the local ecological environment, and that ecological construction through mountain closure and reforestation can better utilize ecological resources in functional area, such as establishing ecotourism and ecological agriculture. Furthermore, the spatial spillover effect W×Dvv×Natural coefficient is 0.0006 at the 1% important level in Model 6, and the robustness tests from Model 7 to Model 9 are also significantly positive. This shows that TPEFAP can indeed benefit neighboring regions through the channel of natural resource scale, such as the promotion of ecotourism which not only enhances local employment diversity, but attracts neighbors to operate there as well. Thus, it is similar to the practice of Chinese residents of central provinces working along the Yangtze River Delta.

## 4. Conclusions

In this study, we develop an MPI to represent the rural poverty and employ SCM to evaluate the impact of TPEFAP on poverty alleviation to a panel data covering key ecological functional areas of China in 2011–2018 and then utilize the dynamic spatial Durbin model to explore its spatial spillovers and mechanisms. The conclusions are summarized as follows:

- The empirical results of synthetic control method reveal that TPEFAP has a positive effect on MPI in Ningxia, Jilin, Hubei, Yunnan, and Gansu, while MPI improvement effect fluctuates in Qinghai. Robustness test results indicate that MPI development in treated provinces is greater than that of donor pools.
- The empirical results of the spatial effect analysis illustrate that TPEFAP not only increases the MPI of local areas but also has positive spillovers on neighboring areas. In addition, TPEFAP significantly improves short-term MPI, and the direct effect on MPI in local areas (0.02067) is almost the same as that in neighboring areas (0.02699). Hence, it indicates that neighboring areas also benefit from the policy at the beginning of the program. In the long term, TPEFAP improves MPI gradually, and the direct effect (0.06046) is gradually weaker than indirect impact (0.09048) on neighboring areas.
- The empirical results of the impact mechanism analysis show that rural labor structure, rural labor mobility, agricultural productivity, and natural resource scale are indeed potential paths of TPEFAP poverty reduction. Conversely, rural labor structure and labor mobility are currently the most critical paths for TPEFAP to achieve alleviation of ecological poverty. From the spatial lag term coefficients of the interaction between TPEFAP and the mechanism variables, the optimization of labor structure can suppress MPI increases in neighboring areas, while TPEFAP can cause a positive spatial spillover on MPI in neighboring provinces through labor mobility accelerates, agricultural productivity improvement, and natural resource scale expansion.

## 5. Policy Implications

Three following policy implications are put forward in accordance with the results above:

- Understanding the importance of TPEFAP for poverty alleviation. The government should promote construction of ecological function areas nationwide and perfect the details of TPEFAP policy and eco-compensation since TPEFAP is effective in improving poverty. The government can then strengthen poverty identification mechanisms at the macro- and micro-levels in the post-poverty alleviation era. Specifically, at the micro level, local governments should make a solid and detailed record of the poor, prioritize eco-logical poverty reduction, and provide targeted support to farmers struggling with multidimensional poverty. Conversely, at the macro level, governments should clarify the main contradictions causing poverty in the region, fully consider local industrial development and economic development patterns, allocate resources rationally, formulate ecological poverty reduction development, and implementation plans in sub-regions, while concentrating on a win–win situation of poverty reduction and environmental sustainability.
- Refining the framework for poverty reduction spillovers. The Chinese government should incorporate the spatial aggregation effect of poverty reduction into the future framework of TPEFAP. For national key ecological function areas with diffusion effects, they should adopt more effective regional synergistic policies for mutual assistance and promote the coordinated development of multidimensional poverty improvement among regions, thus creating positive spatial economic benefits.
- Reinforcing TPEFAP’s channels for poverty reduction. On the one hand, the channel of the scale of natural resources has a little overall effect. Therefore, in the future, the government should make use of the natural resource advantage possessed by poor areas to vigorously develop eco-industries and strengthen its policy effectiveness. Specifically, from the actual situation of different regions, the development mode of ecological agriculture and breeding industry for each region is formulated based on the principle of “forestry as appropriate, fishing as appropriate”. On the other hand, to further optimize the structure of rural labor and increase the proportion of “farmers to workers”, the government should create special agricultural labor training institutions, strengthen labor skills training for farmers, and coordinate use of ecological jobs such as “ecological ranger” jobs to transform the proportion of “farmers to workers” actively. Additionally, the government also should promote ecological migration for farmers located in resource-poor functional areas, which will create jobs and accelerate urbanization of functional areas.

There are still some shortcomings in this study.

Firstly, our selection of certain variable measures is not optimal due to data availability. For example, the urbanization rate to measure the variable *Urban*. In fact, it makes more sense to use the number of rural to urban migrants to calculate rural labor mobility. Secondly, although we have explored several impact paths that can have effects on multidimensional poverty, there are many channels through which the ecological compensation system affects poverty, and we cannot exhaust each of them due to limited space. These shortcomings will be studied in the future.

## Figures and Tables

**Table 1:** Indicator system for assessment of MPI.

Index Dimension	Sub-Dimension	Indicators
Multidimensional Poverty Index (MPI)	Education	Illiteracy or semi-literacy rate of rural residents over 15 years old, Number of primary and secondary school students, Total number of rural teachers;
	Medical	Number of village clinic staff per thousand rural residents, Number of beds in hospitals and health centers per capita, Rural subsistence allowances;
	Economy	Engel coefficient of rural residents, Farmer’s per capital income, Rural per capital housing area, Rural Credit Cooperative Deposit and Loan;
	Ecology	Cultivated area per thousand agricultural population, Total crop production

**Table 2:** Donor pool weights.

Province	Ningxia	Gansu	Yunnan	Qinghai	Hubei	Jilin
Beijing	0.002	0.000	0.000	0.000	0.009	0.022
Tianjin	0.001	0.000	0.000	0.000	0.008	0.132
Hebei	0.001	0.000	0.000	0.000	0.005	0.034
Shanxi	0.100	0.000	0.000	0.000	0.124	0.105
Inner Mongolia	0.001	0.000	0.000	0.000	0.002	0.033
Liaoning	0.001	0.000	0.000	0.000	0.005	0.022
Heilongjiang	0.258	0.000	0.000	0.000	0.100	0.213
Shanghai	0.000	0.028	0.000	0.000	0.027	0.010
Jiangsu	0.075	0.000	0.000	0.367	0.003	0.014
Zhejiang	0.001	0.000	0.000	0.000	0.009	0.010
Anhui	0.000	0.000	0.000	0.000	0.082	0.012
Fujian	0.001	0.000	0.000	0.000	0.010	0.011
Jiangxi	0.002	0.000	0.000	0.000	0.007	0.013
Shandong	0.064	0.000	0.000	0.060	0.004	0.241
Henan	0.001	0.164	0.000	0.000	0.006	0.021
Hunan	0.001	0.000	0.000	0.000	0.007	0.012
Guangdong	0.001	0.000	0.885	0.000	0.008	0.010
Guangxi	0.001	0.000	0.000	0.000	0.006	0.013
Hainan	0.118	0.081	0.000	0.000	0.325	0.006
Chongqing	0.002	0.000	0.000	0.243	0.236	0.011
Sichuan	0.242	0.000	0.000	0.000	0.004	0.015
Guizhou	0.127	0.679	0.063	0.331	0.005	0.008
Shanxi	0.001	0.000	0.000	0.000	0.005	0.016
Xinjiang	0.001	0.048	0.052	0.000	0.005	0.017

Note: The first row lists the names of TPEFAP implementation provinces, and the control group provinces are listed in the first left column. Taking Qinghai as an example, the composite Qinghai includes Jiangsu (36.7%), Shandong (6%), Chongqing (24.3%), and Guizhou (33.1%), each of which has a weight of 1.

**Table 3:** The direct effect estimation results of dynamic spatial Durbin models.

Variables	OLS	FE	SYS-QML	GSPA 2SLS	QML
	Model 1	Model 2	Model 3	Model 4	Model 5
L.MPI			0.535 *** (0.065)		0.647 *** (0.044)
Labor	0.190 (0.124)	0.279 (0.285)	0.184 (0.152)	−0.021 (−0.141)	−0.029 * (−0.130)
Urban	0.220 (0.151)	0.255 (0.242)	0.009 (0.092)	−0.034 (−0.095)	0.004 (0.085)
ATFP	−0.070 (−0.058)	−0.077 *** (−0.018)	−0.024 (−0.019)	−0.003 (−0.020)	0.020 * (0.017)
Natural	0.040 *** (0.005)	−0.00001 (0.000)	−0.000002 (0.000)	0.00005 (0.000)	−1.54 × 10^−6^ (0.000)
lngov	−0.110 *** (0.023)	0.059 *** (0.020)	0.022 (0.013)	0.035 ** (0.014)	0.007 * (0.014)
lnpgdp	0.250 *** (0.026)	0.049 (0.045)	0.044 * (0.025)	−0.028 (−0.024)	−0.162 (−0.024)
lninvest	−0.020 (−0.012)	−0.003 (−0.013)	0.005 (0.010)	0.007 (0.006)	0.003 ** (0.005)
Dvv	0.010 (0.012)	0.026 ** (0.010)	0.009 * (0.005)	0.031 *** (0.007)	0.020 *** (0.006)
W×Dvv				0.070 *** (0.015)	0.015 * (0.013)
${}_{-}Cons$	−1.390 *** (−0.177)	−0.733 *** (−0.243)	−0.514 *** (−0.148)		
$R^{2}$	0.440	0.842	0.893	0.0620	0.900
Log-likelihood				935.636	439.181
$\sigma^{2}$				0.0005	0.047
N	390	390	360	390	360

Note: L. Represents time lag term. W. Represents space lag term. The prefix “ln” before the explanatory variables denotes taking the logarithmic form. ***, **, and * indicate significance at the level of 1%, 5%, and 10%, respectively. Figures in () are the t-values or z-values of the coefficients. Due to space limitations, the spatial lag coefficient of some variables is not listed in the table. Interested readers can ask the author for it.

**Table 4:** Decomposition effects of dynamic spatial Durbin model in short- and long-term.

Variable	Short-Term			Long-Term		
	Direct Effect	Indirect Effect	Total Effect	Direct Effect	Indirect Effect	Total Effect
Dvv	0.02067 *** (0.006)	0.02699 ** (0.013)	0.04766 *** (0.016)	0.06046 *** (0.019)	0.09048 * (0.046)	0.15094 *** (0.056)

Note: ***, **, and * indicate significance at the level of 1%, 5%, and 10%, respectively. Figures in () are the t-values or z-values of the coefficients.

**Table 5:** Influence mechanism of TPEFAP on MPI.

Variables	Model 6	Model 7	Model 8	Model 9
Labor	−0.305 (−7.68)	−0.452 *** (−11.23)	−0.742 *** (−28.56)	−0.407 *** (−15.65)
Urban	0.236 *** (9.23)	0.445 *** (17.14)	0.742 *** (28.56)	0.407 *** (15.65)
ATFP	0.127 *** (6.83)	0.082 *** (4.36)	0.119 *** (6.34)	0.114 *** (6.05)
Natural	0.0002 *** (8.00)	0.0004 *** (16.86)	0.0007 *** (28.19)	0.0004 *** (16.17)
W×Labor	1.703 (14.68)	1.637 *** (13.87)	2.730 (22.96)	2.276 *** (18.81)
W×Urban	0.710 *** (9.55)	0.279 *** (3.72)	0.208 *** (2.76)	0.967 *** (12.66)
W×ATFP	0.478 *** (10.04)	0.223 *** (4.63)	0.515 *** (10.60)	0.383 *** (7.88)
W×Natural	−0.0003 *** (−3.30)	−0.0006 *** (−7.38)	−0.001 *** (−12.70)	0.00006 (0.65)
Dvv×Labor	−1.163 *** (−14.18)	−3.966 *** (−29.67)	−7.307 *** (−47.69)	−4.939 *** (−31.63)
Dvv×Urban	0.127 *** (2.34)	2.302 *** (15.03)	5.032 *** (28.98)	3.599 *** (20.80)
Dvv×ATFP	−0.361 *** (−8.52)	0.031 (0.67)	0.050 (1.07)	0.0002 (0.00)
Dvv×Natural	0.0002 *** (3.03)	0.0007 *** (9.14)	0.0001 (1.46)	0.0004 *** (4.88)
W×Dvv×Labor	3.188 *** (15.54)	14.660 *** (42.43)	19.410 *** (53.88)	17.140 *** (45.44)
W×Dvv×Urban	0.922 *** (7.47)	14.250 *** (34.54)	14.410 *** (34.73)	15.760 *** (36.47)
W×Dvv×ATFP	1.261 *** (14.44)	0.123 (1.32)	0.289 ** (2.88)	0.848 (7.57)
W×Dvv×Natural	0.0006 *** (4.38)	0.001 *** (9.80)	0.001 *** (7.32)	0.0009 *** (5.49)
ρ	0.457 *** (10.81)	1.359 *** (31.49)	2.659 *** (61.53)	0.763 *** (17.80)

Note: *** and ** indicate significance at the level of 1% and 5%, respectively. Figures in () are the t-values or z-values of the coefficients. Additionally, to test the robustness, we added control variables such as economic development (*Pgdp*), government expenditure (*Gov*), and rural investment level (*Invest*) to Models 7 to 9 of Table 5 in order. Due to space limitation, only the estimated results of mechanism variables (Labor, Urban, ATFP, and Natural) and their interactions with policy dummy variables are reported in the table. If readers are interested in the rest of the results, ask the author for them.

## Data Availability

Not applicable.

## Appendix A

**Table A1:** The Evolutionary History of TPEFAP.

Year	Allocation Method	Policy Title	Fund Usage
2009	A province’s subsidies = (∑ standard fiscal expenditures of municipal and county governments included in the pilot area in the province − ∑ standard fiscal revenues of municipal and county governments included in the pilot area in the province) × (1 − balanced transfer coefficient of the province) + special expenditures of municipal and county governments included in the pilot area for ecological and environmental protection × subsidy coefficient of a province	“*Transfer Payments for National Key Ecological Function Areas (Pilot)*”	1. Pollution Control 2. Ecological Construction
2011	A province’s subsidies = ∑ the standard financial gap between the government of the city and county included in the scope of transfer payments × the subsidy coefficient + the special expenditure on ecological and environmental protection of the government of the city and county included in the scope of transfer payments + subsidies for prohibited development zones + provincial guiding subsidies	“*Transfer Payments for National Key Ecological Function Areas*”	1. Pollution Control 2. Ecological Construction 3. Improving farmers’ Livelihoods
2012	A province’s subsidies = ∑ the standard financial gap between the income and expenditure of the counties belonging to the national key ecological function areas such as restricted development in the province × subsidy coefficient + subsidies for prohibited development areas + guiding subsidies + subsidies for the pilot work of ecological civilization demonstration projects	“*Transfer payments from the Central Government to the Local States for Key Ecological Function Areas for 2012*”	1. Pollution Control 2. Ecological Construction 3. Improving farmers’ Livelihoods
2016	A province’s subsidies = key subsidies + subsidies for prohibited development + guiding subsidies ± reward and punishment funds. In calculating subsidies, consider the special expenditures for environmental and ecological protection in each place as well as the employment of poor people as ecological protection personnel	“*Transfer payments from the Central Government to the Local States for Key Ecological Function Areas for 2016*”	1. Ecological Poverty Alleviation 2. Pollution Control 3. Ecological Construction 4. Ecological Migration
2017	A province’s subsidies = key subsidies + subsidies for prohibited development + guiding subsidies ± reward and punishment funds+ forest ranger subsidies	“*Transfer payments from the Central Government to the Local States for Key Ecological Function Areas for 2017*”	1. Ecological Poverty Alleviation 2. Pollution Control 3. Ecological Construction 4. Ecological Migration
2018	Based on the 2017 method, expand the focus of subsidies to the Yangtze River Economic Belt, “three regions and three states” and other deep poverty areas	“*Transfer payments from the Central Government to the Local States for Key Ecological Function Areas for 2018*”	1. Ecological Poverty Alleviation 2. Pollution Control 3. Ecological Construction 4. Ecological Migration

**Table A2:** Variable descriptive statistics.

Variables	Symbol	Definition	Mean	St. Dev.	Max	Min
Poverty improvement	MPI	Entropy weight method is used to construct	0.368	0.117	0.764	0.121
Policy dummy	Dvv	TPEFAP implementation year and region prevail	0.123	0.329	1.000	0.000
Rural non-farm employment	Labor	The ratio of labor force to total population in primary industry	0.207	0.085	0.403	0.016
Rural labor mobility	Urban	Ratio of urban household population to total regional population	0.541	0.136	0.896	0.275
Agricultural total factor productivity	ATFP	Measure by Malmquist index method	1.036	0.075	1.451	0.756
Natural resources	Natural	Per capita afforestation area	58.980	66.200	350.700	0.303
Government expenditure	Gov	financial expenditure per capita	8.178	0.790	10.290	6.297
Economic development	Pgdp	GDP per capita	10.490	0.599	11.850	8.657
Rural investment level	Invest	rural fixed asset investment per capita	7.067	0.644	8.453	4.227

**Table A3:** Global Moran’s index of MPI.

Year	I	P	Year	I	P
2006	0.184 ***	0.000	2013	0.141 ***	0.000
2007	0.177 ***	0.000	2014	0.117 ***	0.000
2008	0.169 ***	0.000	2015	0.122 ***	0.000
2009	0.165 ***	0.000	2016	0.113 ***	0.000
2010	0.157 ***	0.000	2017	0.115 ***	0.001
2011	0.153 ***	0.001	2018	0.100 ***	0.000
2012	0.149 ***	0.002			

Note: *** indicates significance at the level of 1%.

**Table A4:** Test results related to model selection.

Test Type	Null Hypothesis	Statistics	Results
LM test	SEM	49.722 ***	SDM
	Robust SEM	0.605	
	SAR	794.533 ***	
	Robust SAR	745.417 ***	
Hausman test	Random effect	123.500 ***	Fixed effect
Wald test	SDM can be simplified to SEM or SAR	27.360 **	SDM
LR test	SDM can be simplified to SEM or SAR	25.390 ***	SDM
		25.100 ***	

Note: *** and ** indicate significance at the level of 1%, 5%, and 10%, respectively.
